# Seroepidemiology of *Burkholderia pseudomallei*, Etiologic Agent of Melioidosis, in the Ouest and Sud-Est Departments of Haiti

**DOI:** 10.4269/ajtmh.18-0352

**Published:** 2018-09-17

**Authors:** Thomas A. Weppelmann, Michael H. Norris, Michael E. von Fricken, Md. Siddiqur Rahman Khan, Bernard A. Okech, Anthony P. Cannella, Herbert P. Schweizer, Daniel C. Sanford, Apichai Tuanyok

**Affiliations:** 1 Herbert Wertheim College of Medicine, Florida International University, Miami, Florida;; 2 Emerging Pathogens Institute, University of Florida, Gainesville, Florida;; 3 Department of Infectious Diseases and Immunology, College of Veterinary Medicine, University of Florida, Gainesville, Florida;; 4 Department of Global and Community Health, George Mason University, Fairfax, Virginia;; 5 Department of Environmental and Global Health, College of Public Health and Health Professions, University of Florida, Gainesville, Florida;; 6 Department of Medicine, College of Medicine, University of Florida, Gainesville, Florida;; 7 Department of Molecular Genetics and Microbiology, College of Medicine, University of Florida, Gainesville, Florida;; 8 Battelle Biomedical Research Center, Columbus, Ohio

## Abstract

*Burkholderia pseudomallei*, the etiological agent of melioidosis, has been hypothesized to be endemic throughout the Caribbean, including the impoverished nation of Haiti. However, because of the protean clinical manifestations, presence of asymptomatic infections, and limited medical diagnostic capacity, the identification of active melioidosis cases remains challenging. A seroepidemiological study was conducted using a novel enzyme-linked immunosorbent assay (ELISA) to detect antibodies toward *B. pseudomallei* in the native population. The performance of an indirect ELISA with purified lipopolysaccharide (LPS) from *B. pseudomallei* was evaluated using serum collected from rhesus macaques exposed to aerosolized *B. pseudomallei*. After optimization, serum collected from asymptomatic population members (*n* = 756) was screened for polyvalent (immunoglobulin M [IgM]/ immunoglobulin G [IgG]/ immunoglobulin A) and monoclonal (IgG or IgM) immunoglobulins against *B. pseudomallei* LPS. The population seroprevalence was 11.5% (95% confidence interval [CI]: 9.2, 13.8) for polyvalent immunoglobulins, 9.8% (95% CI: 7.7, 11.9) for IgG, and 1.7% (95% CI: 0.8, 2.6%) for IgM. The seroprevalence was not significantly different by gender (*P* = 0.16), but increased significantly (*P* < 0.001) with age, yielding an estimated annual seroconversion rate of 1.05% (95% CI: 0.81, 1.3). The detection of both recent (IgM+) and previous (IgG+) exposure to *B. pseudomallei* provides serological evidence that melioidosis is endemic in Haiti.

## INTRODUCTION

Originally discovered in 1911, melioidosis has gained attention over the past decade because of its possible use as a weapon of bioterrorism.^1–3^ The etiological agent, *Burkholderia pseudomallei*, is a nonsporulating, Gram-negative betaproteobacterium that occurs naturally in surface water and soils throughout tropical and subtropical climates.^4^ Melioidosis is a febrile illness characterized by abscess formation and subsequent hematogenous spread of bacteria to virtually any host organ, initiating most commonly in the lungs.^5^ The clinical manifestations arise days to years after inhalational or percutaneous exposure and include acute suppurative soft tissue infections, acute pulmonary infections, chronic localized infections, and often fulminant septicemia.^6^ Although serological studies suggest most infections are symptomatic, the wide range of clinical manifestations and inherent antibiotic resistance complicate timely diagnosis and effective treatment, leading to mortality rates that can exceed 80%.^7^ Historically, Southeast Asia and Northern Australia were considered the main endemic foci; however, case reports from the Indian subcontinent, Africa, and the Americas have demonstrated a much wider geographic range.^8^ The recent discovery of autochthonous cases in Puerto Rico has led to the hypothesis that melioidosis could be endemic throughout the Caribbean, including the impoverished nation of Haiti.^9^ Nevertheless, because of the protean clinical manifestations, presence of asymptomatic infections, and minimal medical diagnostic capacity, the identification of active cases remains challenging. Therefore, a large serological study was conducted to investigate the potential for human exposure to *B. pseudomallei* in Haiti.

## MATERIAL AND METHODS

### Development of *B. pseudomallei* lipopolysaccharide (LPS) enzyme-linked immunosorbent assay (ELISA).

#### Burkholderia pseudomallei LPS antigen preparation.

An indirect ELISA was developed to detect antibodies toward LPS from the outer membrane of *B. pseudomallei*. Type A LPS was purified from a derivative of *B. pseudomallei* Bp82, an attenuated strain,^10^ using a protocol modified from Lam and others.^11^ Strain Bp82 Δ*wcb*, a capsular polysaccharide (CPS) mutant was grown on 10 plates of tryptic soy agar (TSA) agar for 72 hours. Bacterial lawns were scraped with a plate spreader and suspended in phosphate buffered saline (PBS). Bacterial suspensions were incubated at 110°C for 15 minutes in 2 mL gasketed microcentrifuge tubes. Phenol was added to the lysed solution to a final concentration of 50% and 10% volume of the resulting mixture was plated on TSA to ensure sterility. After sterility verification, samples were gently stirred for 30 minutes at 60°C on a hot plate before dialysis with 12–14 kDa molecular weight cutoff tubing against distilled water for 3–5 days to completely remove phenol. Dialyzed samples were treated with DNase I for 2 hours, RNase H for 2 hours, and Proteinase K overnight, then further purified as previously described.^12^ After lyophilization, dry weights were determined, the residues were resuspended in 2 mL of endotoxin-free water, and diluted to measure endotoxin units using the Pierce limulus amebocyte lysate assay according to manufacturer’s instructions. The resulting crude preparation was separated using a liquid chromatography system (AKTA Purifier; GE Healthcare, Pittsburgh, PA), with a HiPrep Sephacryl S-300 high-resolution column and refractive index detector (Agilent) to fractionate the LPS. The purified LPS was lyophilized, reconstituted in endotoxin-free water to a stock concentration of 100 mg/mL, and frozen at −80°C for future use.

#### Validation of LPS ELISA using previously exposed nonhuman primate serum.

Positive-control serum was obtained from a previous study of nonhuman primates exposed to *B. pseudomallei*, which was conducted at Battelle (in preparation). Briefly, seven rhesus macaques (*Macaca mulatta*) were exposed to a head-only aerosol challenge with 50 (*n* = 3), 100 (*n* = 3), or 500 (*n* = 2) colony-forming units of *B. pseudomallei* strain 1026b. Serum was collected from each animal 7 days before exposure, and at days 7, 14, 21, and 28 post-exposure. Aliquots of serum collected on days −7 (pre-exposure), 7, and 14 were pooled from surviving animals and used as a positive control to determine the appropriate antigen coating concentration (1, 0.5, 0.25, 0.125, and 0.0625 μg/mL) and serum dilution (1:200, 1:400, 1:800, 1:1,600, 1:3,200, 1:6,400, 1:12,800, and 1:25,600). After optimization (Supplemental Figure 1), samples from each animal were analyzed using the LPS ELISA over the course of the infection (7–28 days). The changes in IgM, IgG, and polyvalent immunoglobulins (Ig) (IgM/IgG/IgA) were compared between the pre-exposure and for days 7, 14, 21, and 28 postexposure, using one-way analysis of variance, with a fold increase calculated by dividing the 28-day absorbance values by the pre-exposure absorbance values.

#### Burkholderia pseudomallei LPS ELISA protocol.

High-affinity 96-well polystyrene ELISA plates (Immulon4 HBX; Thermo Scientific, Waltham, MA) were coated with 100 μL of purified LPS at a concentration of 1 μg/mL. After overnight incubation at 25°C, the antigen solution was discarded and the plates were washed three times with washing solution (1% PBS, 0.05% Tween) and blocked for 1 hour with 300 µL of blocking solution (5% nonfat skim milk containing 1% PBS and 0.5% Tween). The blocking solution was discarded and 100 μL of human or nonhuman primate serum diluted 1:1,000 in blocking solution was added. After incubation at 25°C for 1 hour, the primary antibody solution was discarded and the plates were washed five times with washing solution. Horseradish peroxidase (HRP)–conjugated goat antihuman (or anti-monkey) polyvalent immunoglobulin (IgG/IgM/IgA; Thermo Scientific) and HRP-conjugated goat antihuman (or anti-monkey) immunoglobulins G and M (Sigma-Aldrich, St. Louis, MO) were diluted 1:2,000 in blocking solution and 100 μL was added to each well. After incubation for 1 hour at 25°C, the secondary antibody solution was discarded and the plates were washed five times with washing solution and 100 μL of 3, 3′,5,5′-Tetramethylbenzidine (TMB) substrate (1-Step Ultra TMB-ELISA; Thermo Scientific) was added to each well. Reactions were allowed to develop in the dark for 15 minutes before the addition of 100 μL of 1 M phosphoric acid to stop the reaction. The absorbance of each sample was measured at 450 nm using a spectrophotometer (Epoch plate reader; Biotek, Winooski, VT). A subset of human and nonhuman primate samples with absorbance values greater than the 99^th^ percentile from the poly Ig ELISA were further characterized by reactivity toward purified CPS, purified LPS, and whole-cell lysate by Western blot as described previously.^13^ The CPS was harvested and purified from *B. pseudomallei* Bp82 Δ*wbiI*, an O-antigen mutant made in this study using the same methodology as previously described; whole-cell lysate was from *B. pseudomallei* Bp82.^12^

### Study location and enrollment of human participants.

Ethical approval to conduct this research was obtained from the Haitian-based ethical review committee and the University of Florida (UF) Institutional Review Board (IRB #201300023). Asymptomatic (nonfebrile) community members (*n* = 756) from the Ouest and Sud-Est departments of Haiti were enrolled between February and May 2013 using a convenience sample that included native Haitians aged between 2 and 80 years old (average age 13.5 years). Recruitments consisted of students attending a primary school and family members of patients attending local clinics in Gressier (*n* = 575) and Jacmel (*n* = 150), as well as individuals seeking routine care at a mobile health clinic in the rural community of Chabin (*n* = 31). After obtaining consent from participants or their guardian, approximately 3 mL of venous blood was collected in serum separation tubes (BD Vacutainer, Franklin Lakes, NJ) and immediately centrifuged at 6,000 rotations per minute for 2 minutes. Serum samples were stored at −80°C in the UF field laboratory in Gressier, Haiti, and shipped to the UF-Emerging Pathogens Institute, in Gainesville, Florida, for serological analyses.

### Statistical analyses of seroprevalence by demographic factors.

The human samples were first screened for total anti–*B. pseudomallei* LPS (IgM/IgG/IgA) and then individually for anti–*B. pseudomallei* LPS IgM or IgG. A single absorbance value was calculated for each participant by taking the average measurement from serum samples run in triplicate using the anti–*B. pseudomallei* LPS ELISA. Because no cases of melioidosis have been reported from Haiti and this study has a relatively large sample size (*n* = 756), it was assumed that the majority of the population would not have antibodies toward *B. pseudomallei* LPS. In that case, the absorbance values from seronegative population members would follow a Gaussian distribution defined by sample mean $\left(\hat{\text{μ}}\right)$ and sample standard deviation $\left(\hat{\text{σ}}\right)$. An absorbance threshold (λ_pos_) was set at least three sample standard deviations from the mean of the suspected seronegative population to exclude ≥ 99.7% of seronegative members from being classified as seropositive. The number and proportion of seropositive participants for anti–*B. pseudomallei* IgM, IgG, and IgM or IgG (either) was tabulated by gender, age group, and enrollment location before testing statistical associations between demographic factors using simple logistic regression (Stata v 13 College Station, TX). The cross-sectional seroprevalence data were also used to estimate the seroconversion rate as previously described.^14^

## RESULTS

### *Burkholderia pseudomallei* antibody detection using exposed nonhuman primate serum.

**Figure 1.**
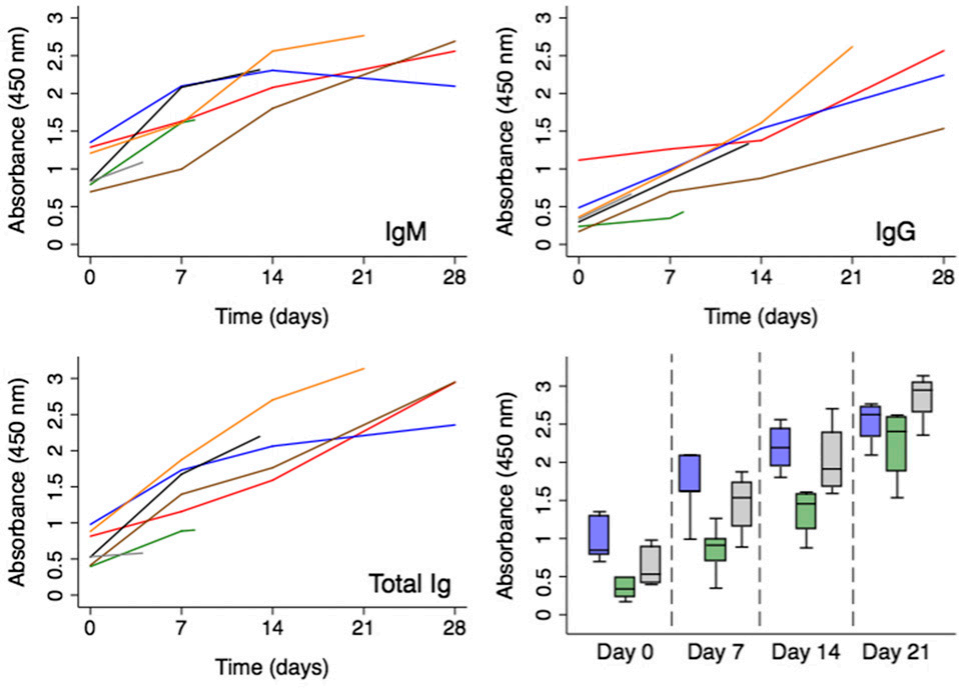
Non-human primate immunological response and survival after inhalational exposure to *Burkholderia pseudomallei.* The immunological responses of nonhuman primates (*n* = 7) exposed to *B. pseudomallei* 1026b were measured over the course of infection. The absorbance values (λ = 450 nm) from the lipopolysaccharide enzyme-linked immunosorbent assay obtained using polyvalent (IgG/IgM/IgA), IgM, and IgG secondary antibodies are presented by study animal (various color lines) for serum collected at 7 days pre-exposure and 7, 14, 21, and 28 days postexposure along with the box plot of the data aggregated from animals pre-exposure (time = 0), 7, 14, and after 21 days postexposure for IgM (blue boxes), IgG (green boxes), and total Ig (gray boxes).

**Figure 2.**
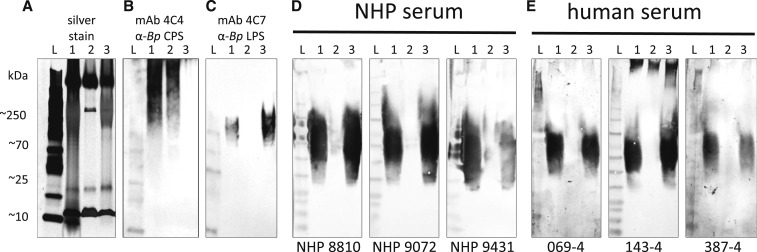
Western blots of NHP and human immune response to *Burkholderia pseudomallei* lipopolysaccharide (LPS) and capsular polysaccharide (CPS). Western blotting was used to confirm the presence of anti–*B. pseudomallei* polyvalent immunoglobulins (IgM/IgG/IgA) in NHP and human sera. Total antigens from heat-killed *B. pseudomallei* Bp82 (in lane 1), the purified CPS in lane 2, and the purified LPS in lane 3 were used to detect the polyvalent Ig responses in NHP and human sera. The antigens were resolved by sodium dodecyl sulfate polyacrylamide gel electrophoresis and silver stained (**A**) for visualization, and confirmed for their antigenicity by detecting with the CPS-specific mAb 4C4 (**B**), the LPS-specific mAb 4C7 (**C**), and humoral immunity responses in the NHP sera (**D**) and the human sera (**E**). We noted that the coded sera from the enzyme-linked immunosorbent assay–positive NHPs and humans showed the presence of the anti-LPS polyvalent immunoglobulins. Lane L, the pre-stained protein ladder.

The exposure of rhesus macaques to an aerosol challenge resulted in pathological lesions consistent with inhalational melioidosis and abscesses that yielded *B. pseudomallei* on culture.^15^ The immunological responses measured using the LPS ELISA are presented over the course of infection for individual animals (*n* = 7) and aggregated for all surviving animals on days −7 (prior to exposure), 14, and 21 postexposure (Figure 1). Compared with serum collected pre-exposure, there was a significant increase in IgM concentration (*P* = 0.012) by day 7 postexposure, followed by a significant increase in IgG concentration (*P* = 0.004) by day 14 postexposure; the polyvalent Ig (IgA/IgM/IgG) concentration significantly increased (*P* = 0.004) by day 7 postexposure. By day 21 postexposure, the 57.1% (4/7) of animals that survived had a significant increase in absorbance using the LPS ELISA, with an average increase in absorbance of 2.5 fold for IgM, 5.2 fold for IgG, and 4.4 fold for polyvalent (IgA/IgM/IgG) immunoglobulins (Supplemental Table 1). The immune responses toward purified CPS, LPS, and whole-cell lysate were further characterized by Western blot (Figure 2). There was little to no reactivity toward the CPS. By contrast, the ladder pattern observed with purified LPS indicated that it was highly immunogenic.

### Seroprevalence of anti–*B. pseudomallei* immunoglobulins in human samples.

**Figure 3.**
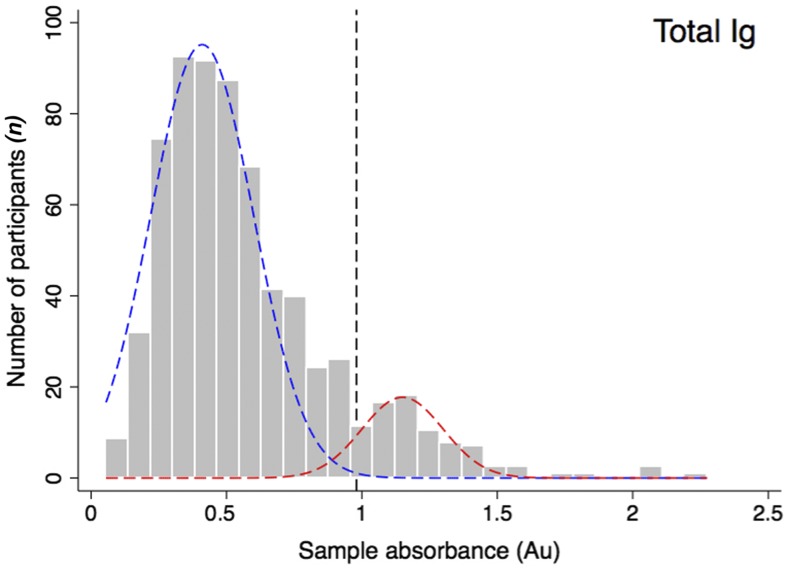
Distribution of total anti–*B. pseudomallei* lipopolysaccharide (LPS) immunoglobulins (polyIg). The distribution of absorbance values (λ = 450 nm) from human serum screened for the presence of polyIg (IgG/IgM/IgA) antibodies using the *B. pseudomallei* LPS enzyme–linked immunosorbent assay are presented. The suspected seronegative population (blue dotted line), the suspected seropositive population (red dotted line), and the absorbance threshold (black dotted line) are overlaid on all absorbance values (gray bars). Au = absorbance units. This figure appears in color at www.ajtmh.org.

**Figure 4.**
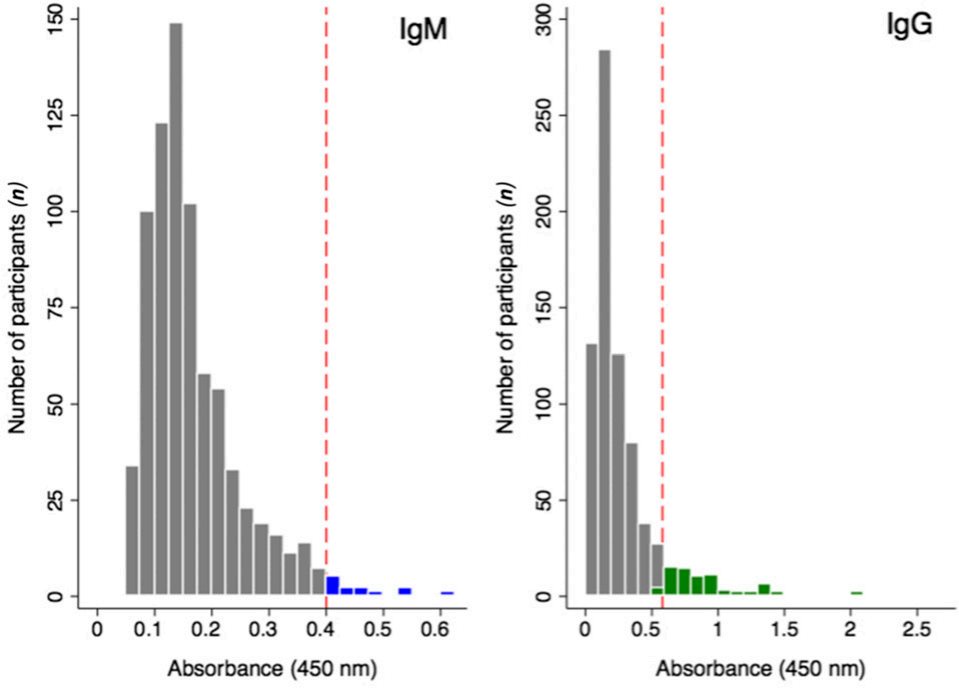
Distribution of anti–*Burkholderia pseudomallei* lipopolysaccharide (LPS) immunoglobulins G and M. The distributions of absorbance values (λ = 450 nm) from human serum screened for the presence of IgG or IgM antibodies using the *B. pseudomallei* LPS enzyme-linked immunosorbent assay are presented. The absorbance thresholds for classification of samples as seropositive (red dotted line), the seropositive populations for IgG (green) and IgM (blue) are presented along with seronegative population members (gray bars). This figure appears in color at www.ajtmh.org.

**Figure 5.**
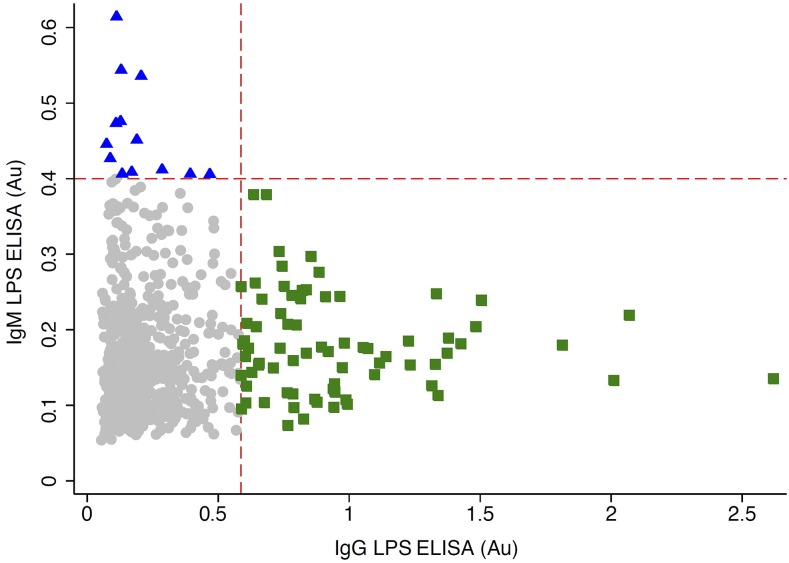
Relationship between anti–*Burkholderia pseudomallei* lipopolysaccharide (LPS) immunoglobulins G and M. A scatter plot of the absorbance values (λ = 450 nm) from human serum screened for the presence of IgG or IgM antibodies using the *B. pseudomallei* LPS-enzyme-linked immunosorbent assay (ELISA) is presented. The absorbance thresholds for classification of samples as seropositive (red dotted line), the seropositive populations for IgG (green squares) and IgM (blue triangles) are presented along with seronegative population members (gray circles). Au = absorbance units. This figure appears in color at www.ajtmh.org.

The absorbance values from the initial ELISA screening of human samples for polyvalent (IgM/IgG/IgA) antibodies toward *B. pseudomallei* LPS is presented in Figure 3. The distribution was bimodal, with a major peak at 0.41 and a minor peak at 1.2 absorbance units, representing the means of the suspected seronegative and seropositive population members, respectively. The absorbance values for the seronegative population were approximately normally distributed about $\hat{\text{μ}}$ = 0.41 and $\hat{\text{σ}}$ = 0.19, yielding an absorbance threshold value of λ_pos_ = 0.98. Using this threshold, 11.5% (95% confidence interval [CI]: 9.2, 13.8) were seropositive for polyvalent (IgM/IgG/IgA) antibodies against *B. pseudomallei* LPS. The absorbance values and thresholds for the presence of IgM or IgG antibodies against *B. pseudomallei* LPS are presented in Figure 4. Both distributions were unimodal with population means and sample standard deviations of 0.13 (±0.15) for the IgG and 0.16 (±0.08) for the IgM LPS-ELISA. Using an absorbance threshold of 0.58 for IgG and 0.4 for IgM, 9.7% (95% CI: 7.7, 11.9) of population members were seropositive for anti–*B. pseudomallei* LPS IgG and 1.7% (95% CI: 0.8, 2.6) were seropositive for anti–*B. pseudomallei* LPS IgM antibodies. As presented in Figure 5, the relationship between the absorbance values obtained from the IgG and IgM LPS ELISA revealed evidence of both recent (IgM only) and past exposures (IgG only), but no participants seropositive for both IgM and IgG antibodies.

### Seroprevalence of *B. pseudomallei* by demographic factors.

**Figure 6.**
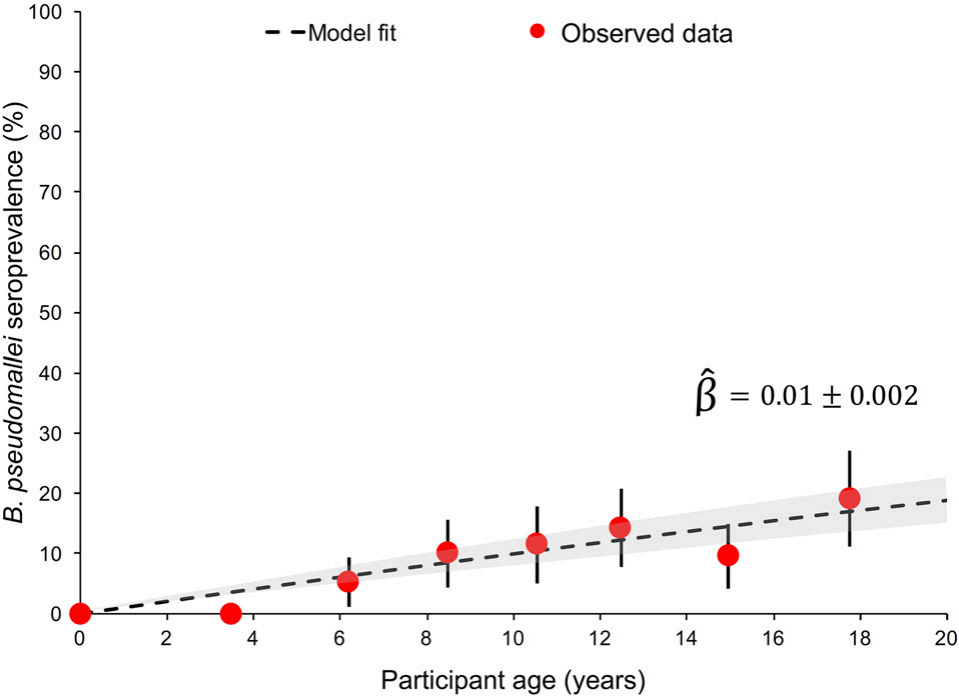
Seroconversion of anti–*Burkholderia pseudomallei* lipopolysaccharide (LPS) immunoglobulins G and M. The age-specific seroprevalence for the presence of either IgM or IgG antibodies toward *B. pseudomallei* LPS antibodies is presented by the average age of participants in that group (red circles) along with the predicted seroprevalence using the reverse catalytic model (black dotted line), the corresponding 95% confidence intervals for the predicted seroprevalence (gray shaded region), and the estimated seroconversion rate $\hat{\text{β}}$ plus or minus the standard error of $\hat{\text{β}}$. This figure appears in color at www.ajtmh.org.

The number of anti–*B. pseudomallei* LPS IgM, IgG-positive samples, and the seroprevalence of samples positive for either immunoglobulin (IgM or IgG) are presented by gender, age group, and enrollment location in Table 1. Females had a higher seroprevalence compared with male community members (12.9% versus 9.6%), but the seroprevalence was not significantly different by gender (*P* = 0.157). Participant age was significantly associated (*P* < 0.001) with an increase in the likelihood of previous exposure to *B. pseudomallei* LPS. Differences were also observed between enrollment sites, where the urban and peri-urban locations (Gressier, 12.2% and Jacmel, 16.0%) had lower rates of previous exposure compared with the rural village of Chabin (29.0%). After adjustment for the age of participants using logistic regression models, no statistically significant differences were identified by enrollment location (*P* > 0.1), with age remaining the only significant predictor of previous exposure (*P* = 0.026). Both the age-specific seroprevalence rates and the annual seroconversion rate estimated by the reverse catalytic model are presented in Figure 6. For participants less than or equal to 20 years of age, the seroconversion rate was 1.05% (95% CI: 0.81, 1.28) of the sample population per year.

**Table 1:** Seroprevalence in native Haitians by demographic factor

Demographic factor		IgM	IgG	Total	Seroprevalence		
	(*n*)	Number positive			(%)	95% CI	
Gender							
Male	314	2	28	30	9.6	6.3	12.8
Female	442	11	46	57	12.9	9.8	16.0
Age group							
< 5	26	0	0	0	0.0	0.0	0.0
6–7	114	0	6	6	5.3	1.1	9.4
8–9	110	3	8	11	10.0	4.3	15.7
10–11	96	1	10	11	11.5	5.0	17.9
12–13	112	2	14	16	14.3	7.7	20.9
14–16	115	3	8	11	9.6	4.1	15.0
17–20	94	4	14	18	19.1	11.0	27.3
> 20	89	0	14	14	15.7	8.0	23.4
Location							
Gressier	575	13	41	54	12.2	7.0	11.8
Jacmel	150	0	24	24	16.0	10.1	21.9
Chabin	31	0	9	9	29.0	12.1	46.0
Total	756	13	74	87	11.5	9.2	13.8

CI = confidence interval; LPS = lipopolysaccharide. The seroprevalence of anti–*Burkholderia pseudomallei* LPS immunoglobulins (IgG, IgM, and either IgG or IgM) detected using the LPS enzyme-linked immunosorbent assay are presented by demographic factors (gender, age group, and enrollment location), with the sample size (*n*), number of positive samples, and the seroprevalence (%) for either IgG or IgM with the corresponding 95% CIs.

## DISCUSSION

Traditionally, the indirect hemagglutination assay (IHA) has been regarded as the serological method of reference for melioidosis; however, it lacks a standard antigen preparation and yields only semiquantitative results.^16^ For these reasons, ELISA-based methods have become increasingly popular, yet have also suffered criticism for not being properly validated and/or using crude antigen preparations.^17^ After multiple reports have compared the two methods using serum from culture-confirmed melioidosis patients and non-endemic controls, the emerging consensus is that ELISA using purified antigens has superior sensitivity and specificity compared with ELISA with crude antigen preparations or IHA.^18–20^ In the present study, we chose to validate this LPS ELISA by measuring the development of antibodies toward *B. pseudomallei* using an inhalation melioidosis model in rhesus macaques that closely mimics human infections.^21^ Not only was there a 2.5–5.2-fold increase in antibody concentration over the course of infection (Figure 1, Table 1), but also the antibody responses in humans and nonhuman primates showed similar patterns on Western blot (Figure 2). Contrary to a previous report, the antibodies generated in the nonhuman primates or identified in the human samples were not reactive toward the CPS but were highly reactive toward the LPS.^22^

Haiti has been referred to as probably endemic by multiple review studies, with the ubiquitous citation of a 1982 edition French journal documenting the isolation of *B. pseudomallei* from soil in Haiti.^8,23–25^ Other than a report of a man from the Dominican Republic diagnosed with melioidosis while receiving treatment for lymphoma in Argentina, there are no other reports of melioidosis from the island of Hispaniola.^26^ Even though there has been little direct evidence of melioidosis in Haiti, to our knowledge there has also been no prior active surveillance. Indeed, the results from this study are consistent with human exposure to *B. pseudomallei*. Although a different serological assay was used in the present study, the seroprevalence in this subset of the Haitian population (12.2–29%) was similar to surveys conducted in endemic regions of India (10.2–29%), Vietnam (6.4–31.8%), Malaysia (1.9–15.8%), and northern Queensland, Australia (8–29%).^27–30^ The increase of seroprevalence with age (Figure 6) was nearly identical to patterns observed in a study conducted in an endemic region of Cambodia, where *B. pseudomallei* was isolated from 30% of all soil samples.^31^ If seroprevalence of IgM antibodies or the seroconversion rate estimated by this study are an indicator of the presence of subclinical infections, incidence in this population could be approximately 0.8–2.6% based on the individuals who were seropositive for the anti–*B. pseudomallei* LPS IgM. Nevertheless, until the first autochthonous case is detected, melioidosis will remain by definition non-endemic in Haiti.

If melioidosis were to be discovered in Haiti, whether *B. pseudomallei* was always present or recently introduced would be difficult to determine; although Haiti’s history provides at least one plausible explanation. Between 1650 and the late 1700s, Haiti (then Saint-Domingue) occupied most of the island of Hispaniola and was estimated to have received more than 700,000 slaves from Africa, along with livestock, plants, and whatever microorganisms they carried.^32,33^ This time period coincides with the introduction of the most recent common ancestor of *B. pseudomallei* from Africa to the Americas (1682–1849) estimated by a large-scale phylogeographic study.^34^ Once imported, the tropical climate and non-fastidious growth requirements for *B. pseudomallei* could have facilitated the establishment of environmental reservoirs in the soil; just as the combination of agricultural exposures and lack of adequate hygiene could have provided ideal conditions for transmission to humans. However, until the first isolation and characterization of the causative bacterium in Haiti, the origins of *B. pseudomallei* (if present) will remain a mystery. In the meantime, we hope the results from this study will continue to encourage future investigations of *B. pseudomallei* throughout the tropics and eventually lead to a proper estimation of the global burden of melioidosis.

## LIMITATIONS

Although the seropositive population members in this study were presumably the result of asymptomatic or undiagnosed infections, antibody production after exposure to an antigenically similar microorganism indigenous to Haiti remains a possible explanation. In the present study, we were unable to correlate the presence of IgM antibodies with the presence of disease in this population-based serological survey as samples were analyzed retrospectively and positive individuals were not given medical examination. Likewise, the use of a novel assay might make comparisons of the seroprevalence observed in this study to previous studies conducted in other countries using IHA less valid. We noted that in other endemic countries, non-virulent near neighbors such as *Burkholderia thailandensis* may elicit identical immune responses; however, the occurrence of *B. thailandensis* in Haitian soil is unknown.^35^ Furthermore, using atypical type B LPS as a coating antigen yielded no reactivity toward human samples and no appreciable increase in antibody concentration in nonhuman primates’ exposure to *B. pseudomallei* 1026b, a type A LPS strain. The sample collected was also one of convenience and might not be representative of other departments or subpopulations in Haiti. The prevalence was heterogeneous between locations, but age most highly influenced the overall seroprevalence. In this study, a younger sample population might have underestimated the true level of human exposure to *B. pseudomallei* in Haiti. Finally, the inclusion of serum samples from a non-endemic country for comparison with the Haitian community members would have increased our ability to conclude that serum samples that were three standard deviations from the population mean were actually seropositive.

## CONCLUSION

The findings from this seroepidemiological study demonstrate the potential utility of the LPS ELISA for population-level surveillance and represent the first investigation of antibodies to *B. pseudomallei* in Haiti. After screening a relatively large sample of native Haitians, this study was able to identify serological trends in the sample population consistent with human exposure to *B. pseudomallei*. This study adds to the growing body of research suggesting that *B. pseudomallei* is likely present throughout the Caribbean and support the hypothesis that melioidosis is endemic in Haiti. Hopefully, these findings will encourage environmental sampling to isolate the causative bacterium and increase the level of clinical suspicion for melioidosis in Haiti.

## Supplementary Material

Supplemental table and figure
