# Supplementary material for: Seroepidemiology of Burkholderia pseudomallei, Etiologic Agent of Melioidosis, in the Ouest and Sud-Est Departments of Haiti
Source: Am J Trop Med Hyg. 2018 Sep 17;99(5):1222–8. doi: 10.4269/ajtmh.18-0352 (PMC6221251; doi:10.4269/ajtmh.18-0352)
Supplement: Supplementary file 1 [file tpmd180352.SD1.pdf]

Supplemental Table and Figure

Supplemental Table 1. NHP immunological response post inhalational exposure to *B. pseudomallei*

**One-way ANOVA**		**IgM Absorbance**		**IgG Absorbance**		**Total Ig Absorbance**	
Time (days)	*(n)*	*avg*	*std dev*	*avg*	*std dev*	*avg*	*std dev*
Pre-exposure	7	1.002	0.269	0.430	0.319	0.649	0.238
7	6	1.670	0.405	0.853	0.310	1.454	0.378
14	4	2.187	0.323	1.349	0.329	2.030	0.489
21	4	2.528	0.301	2.241	0.499	2.847	0.339
**F statistic (df = 20)**		21.850		23.460		35.850	
***P* value**		< 0.001		< 0.001		< 0.001	
**Fold-increase**		2.522		5.209		4.384	

The immunological responses of non-human primates (n = 7) exposed to *B. pseudomallei* 1026b was measured over time using the LPS-ELISA. The absorbance values (λ = 450) from the LPS-ELISA obtained using polyvalent (IgG/IgM/IgA), IgM, and IgG secondary antibodies are presented for serum samples collected 7 days pre-exposure, and 7, 14, 21 days post-exposure. The F statistic from the one-way ANOVA (with 20 degrees of freedom) and *P* value are presented with the average increase in absorbance expressed as a fold-increase from pre-exposure to after day 21 post-exposure.

Supplemental Figure 1. LPS-ELISA optimization with non human primate serum

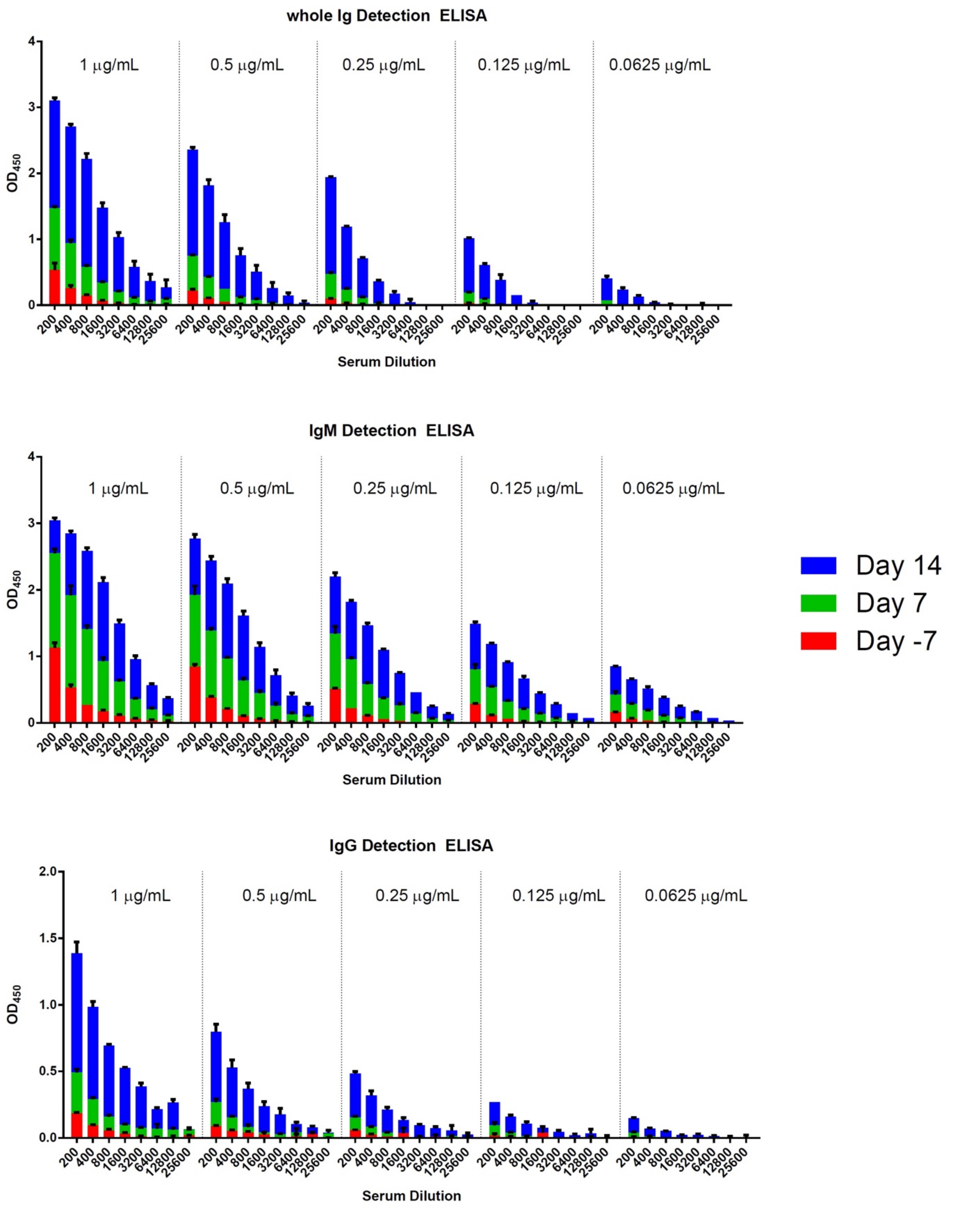

Supplemental Figure 1 Caption: The concentration of LPS used to coat the ELISA plates and serum sample dilutions were optimized over a range of concentrations with positive control serum collected from non-human primates (NHP). The absorbance values (λ = 450) obtained using polyvalent (IgG/IgM/IgA), IgM, and IgG secondary antibodies are presented as stacked bars representing serum samples collected 7 days pre-exposure (red bar), 7 days post-exposure (green bar), and 14 days post-exposure (blue bar).
